# Preventive Aspects of Early Resveratrol Supplementation in Cardiovascular and Kidney Disease of Developmental Origins

**DOI:** 10.3390/ijms22084210

**Published:** 2021-04-19

**Authors:** Chien-Ning Hsu, Chih-Yao Hou, You-Lin Tain

**Affiliations:** 1 Department of Pharmacy, Kaohsiung Chang Gung Memorial Hospital, Kaohsiung 833, Taiwan; cnhsu@cgmh.org.tw; 2 School of Pharmacy, Kaohsiung Medical University, Kaohsiung 807, Taiwan; 3 Department of Seafood Science, National Kaohsiung University of Science and Technology, Kaohsiung 811, Taiwan; chihyaohou@webmail.nkmu.edu.tw; 4 Department of Pediatrics, Kaohsiung Chang Gung Memorial Hospital and Chang Gung University College of Medicine, Kaohsiung 833, Taiwan; 5 Institute for Translational Research in Biomedicine, Kaohsiung Chang Gung Memorial Hospital and Chang Gung University College of Medicine, Kaohsiung 833, Taiwan

**Keywords:** resveratrol, oxidative stress, chronic kidney disease, cardiovascular disease, hypertension, developmental origins of health and disease (DOHaD), gut microbiota, nitric oxide

## Abstract

The increase in the incidence of cardiovascular diseases (CVDs) and kidney disease has stimulated research for strategies that could prevent, rather than just treat, both interconnected disorders. Resveratrol, a polyphenolic compound with pleiotropic biofunctions, has shown health benefits. Emerging epidemiological data supports that early life environmental insults are regarded as increased risks of developing CVDs and kidney disease in adulthood. Conversely, both disorders could be reversed or postponed by shifting interventions from adulthood to earlier stage by so-called reprogramming. The purpose of this review is first to highlight current epidemiological studies linking cardiovascular and renal programming to resulting CVD and kidney disease of developmental origins. This will be followed by a summary of how resveratrol could exert a positive influence on CVDs and kidney disease. This review also presents an overview of the evidence documenting resveratrol as a reprogramming agent to protect against CVD and kidney disease of developmental origins from animal studies and to outline the advances in understanding the underlying molecular mechanisms. Overall, this review reveals the need for future research to further clarify the reprogramming effects of resveratrol before clinical translation.

## 1. Introduction

Non-communicable diseases (NCDs) are the most common causes of death all over the world, accounting for almost two thirds of all global deaths [1]. Cardiovascular disease (CVD) and chronic kidney disease (CKD) are major NCDs. CVD, a cluster of disorders of the heart and blood vessels, accounts for most NCD deaths. Besides, CKD is a major determinant of the adverse health outcomes of NCDs [2]. An estimated roughly 1 in 10 people worldwide may have CKD [3]. CVD and kidney disease are closely interconnected and damage to one organ leads to dysfunction of the other, eventually resulting in the failure of both organs [4]. It is well established in the literature that CVD is a major cause of morbidity and mortality in patients with CKD [5]. On the other hand, heart failure is a common cause of hospitalization with worsening kidney function [6].

Despite recent advances in pharmacological management, both CVD and kidney disease are still increasingly prevalent disorders. Although CVD and kidney disease are most common in older adults, both disorders take their origins from early life, not only childhood and tracing back into the fetal stage too, and progress slowly across the life span [7,8]. The fetal cardiovascular and renal systems are vulnerable to the adverse effects of in utero exposure to environmental insults [7,8]. Now known as the “developmental origins of health and disease” (DOHaD) [9], this theory describes that developmental plasticity accommodates structural changes and functional adaption during organogenesis. In both cardiovascular and renal systems, developmental programming results in endothelial dysfunction, low nephron endowment, fewer cardiomyocytes, stiffer vascular tree, small coronary arteries, aberrant renin–angiotensin system (RAS) and renal sodium handling, and renal dysfunction [7,8,10,11,12,13].

Conversely, programming processes could be theoretically postponed or reversed before clinical disease becomes evident by shifting therapeutic interventions from adult life to earlier stage, so-called reprogramming [14]. Although many mechanisms in which CVD can induce kidney disease, and vice versa, are not yet fully understood, in recent years our understanding of both disorders can originate from early life via common mechanistic pathways has advanced greatly, helping us develop ideal reprogramming strategies to prevent, rather than merely treat, both disorders from happening [14,15,16,17].

Resveratrol is a polyphenol produced naturally by many plants, particularly grape and peanut [18,19]. Numerous studies have demonstrated its wide range of beneficial effects on human health, including CVD and kidney disease [19,20,21]. Much of prior work exploring the therapeutic actions of resveratrol has mainly studied in established CVD and kidney disease.

Through these findings, one may hypothesize that early resveratrol supplementation can serve as a reprogramming strategy to prevent developmental programming of CVD and kidney disease. This review aims to provide insight of resveratrol implicated in cardiovascular as well as renal programming. Firstly, we present the evidence from epidemiological research supporting influences in early life that can program CVD and kidney disease in later life. In the second part, we summarize current knowledge on the beneficial effects of resveratrol in CVD and kidney disease. Finally, we document data on early resveratrol supplementation as a reprogramming strategy to protect adult offspring against kidney disease and CVD of developmental origins.

Our search strategy was designed to retrieve related literature from PubMed/MEDLINE indexed articles. Search terms comprised “cardiovascular disease”, “kidney disease’, “developmental programming”, “DOHaD”, “atherosclerosis”, “heart”, “vascular”, “endothelial dysfunction”, “resveratrol”, “nephron”, “nephrogenesis”, “mother”, “pregnancy”, “gestation”, “offspring”, “progeny”, “reprogramming”, and “hypertension”. We also used the reference lists of identified articles to find additional studies. The last search was made on 20 February 2021.

## 2. Developmental Origins of CVD and Kidney Disease: Human Evidence

Many epidemiological studies in the DOHaD field have concerned CVD [22]. Famine exposure cohorts (i.e., Dutch 1944–1945, Saint Petersburg 1941–1944, and Biafra 1967–1971) indicate that early-life undernutrition is associated with a number of risks factors for CVD, including hypertension, dyslipidemia, obesity, albuminuria, and type 2 diabetes [23,24,25,26]. Another important support for cardiovascular and renal programming came from epidemiological observations showing the associations between early life attributes, in particular low birth weight (LBW) and prematurity, and later CVD and kidney disease [27,28]. In twins, the association between LBW and high blood pressure (BP) is described in childhood [29]. The lighter twins develop arterial narrowing and endothelial dysfunction and are prone to die from ischemic heart disease [29,30].

A reduced nephron number is presumed to be a common risk factor underlying the susceptibility to kidney disease and CVD in adulthood [31]. Nephron is a functional unit of the kidney. Each human kidney contains around one million nephrons, with a 10-fold difference among individuals [32]. During human kidney development, the primitive glomerulus is formed by 9 weeks of gestation and completes at 36 weeks of gestation [33]. Epidemiological studies identify some perinatal risk factors, like LBW, prematurity, gestational diabetes, and maternal obesity as associated with CKD [31,33,34]. Importantly, LBW and prematurity both are related to low nephron endowment [31,33,34].

In addition to reduced nephron, impaired nephrogenesis can cause a wide spectrum of defects in the kidney and urinary tract, namely congenital anomalies of the kidney and urinary tract (CAKUT) [35]. Unlike adults, CAKUT is one of the major causes of CKD in children [36]. As CAKUTs are marked as varying deficits in nephron number, a reduced nephron endowment detected in CAKUT can cause glomerular hyperfiltration, compensatory glomerular hypertrophy, further nephron loss, and accordingly such a vicious cycle leads to CKD progression [37]. A case-control study consisting of 1.6 million infants reported that risk factors for CAKUT consist of prematurity, LBW, maternal thalassemia, male, oligohydramnios or polyhydramnios, gestational diabetes, and first parity [38]. Together, these observations support the notion that LBW and prematurity are major determinants of cardiovascular and renal programming.

A growing body of epidemiological evidence exists regarding environmental influences in early life that can program later CVD and kidney disease, as reviewed elsewhere [7,8,9,10,12,13,14,15,16,22]. These adverse influences include maternal smoking, maternal overnutrition, maternal illness, exposure to medication or environmental toxins, together with undernutrition. There is a positive association of maternal smoking with child obesity, hypertension, and type 2 diabetes, all of which are contributors for CVD [39]. Overnutrition attributed to maternal diabetes or obesity is associated with type 2 diabetes and obesity in offspring, both risk factors for CVD [40,41]. There are also reports showed an association between early-life environmental endocrine-disrupting chemical exposure, like bisphenol, and cardiometabolic traits in childhood [42,43]. Medication uses in pregnancy, like glucocorticoid [44] and non-steroidal anti-inflammatory drugs [45], are also linked to adverse cardiovascular and renal outcomes in offspring. Moreover, several other perinatal risks affecting cardiovascular and renal outcomes in offspring have been reported, such as low gestational hypertension [46], vitamin D intake [47], short-term breastfeeding [48], and excess early postnatal weight gain [49].

However, these epidemiological studies do not allow us to establish direct cause–effect relationships. So, it stands to reason that the uses of animal models to understand which developmental window is decisive for programming, to identify molecular mechanisms behind programmed CVD and kidney disease, and to develop ideal reprogramming strategies.

## 3. Implications of Resveratrol in CVD and Kidney Disease

### 3.1. Resveratrol: Synthesis, Metabolism, and Function

Resveratrol (trans-3,5,4′-trihydroxystilbene) is a well-known phenolic compound from the stilbene family consisting a C6–C2–C6 unit [50]. Sources of resveratrol in food consist of the grapes, blueberries, mulberries, raspberries, and peanuts.

Resveratrol exists as two isomers, namely, trans- and cis-resveratrol. The former one is the more biologically active isomer. In plants, resveratrol is synthesized as a response to damage, stressful conditions, and mechanical injury. Under physiological conditions, glucose is metabolized to 4-coumaroyl-CoA and is combined with malonyl-CoA via stilbene synthase to produce trans-resveratrol. On the other hand, both trans- and cis-isomers can be produced in response to ultraviolet or bacteria exposure. Although trans-resveratrol occurs naturally in grapes, during vinification process trans-resveratrol can be transformed into its cis-form. The trans-resveratrol can be stable for months when protected from light, whereas cis-resveratrol was stable only in neutral pH [51].

Resveratrol is a bioactive molecule with pleiotropic biofunctions on various organ systems. The use of resveratrol as a nutraceutical has been evaluated in a variety of disorders in both animal models and human trials [19,52]. After oral administration, an estimated minimum of 70% of the intake of resveratrol is absorbed [52]. Resveratrol is absorbed passively by diffusion or by forming complexes with intestinal membrane transporters. Sulfation and glucuronidation in the liver are the principal metabolic pathways of resveratrol; accordingly its free form is at very low levels in the circulation [53]. The major forms of resveratrol in the circulation and target organs are sulfate (trans-resveratrol-3,4′-disulfate, trans-resveratrol-3-sulfate, trans-resveratrol-3,5-disulfate) and glucuronide (trans-resveratrol-4′-glucuronide, trans-resveratrol-3-glucoronide) conjugate metabolites. Resveratrol can be rapidly metabolized, with an elimination half-life of 130–180 min [52]. Besides, other resveratrol derivatives like piceatannol and dihydroresveratrol can also be detected in target organs [54,55]. Moreover, gut microbiota is involved in the metabolism of resveratrol by increasing its availability from resveratrol precursors and producing resveratrol derivatives [56]. With a high inter-individual variation, approximately 20–70% and 15–50% absorption of orally ingested resveratrol is reported in humans and rats, respectively [57,58]. To sum up, the bioavailability of resveratrol and its metabolites largely differs from one another, mainly depending on the administration rate and dose, as well as intestinal microbial environment [53].

The multifaceted biological effects of resveratrol include the improvement of endothelial function, antioxidant properties, anti-inflammatory effects, inhibition of platelet aggregation, anti-obesogenic activity, anticarcinogenic properties, anti-atherosclerotic properties, aryl hydrocarbon receptor (AhR) antagonist effects, and restoration of NO bioavailability [18,20,21,25,59]. Resveratrol has been shown to affect multiple molecular targets such as the silent information regulator-1 (SIRT-1), mammalian target of rapamycin (mTOR), nuclear factor-kappa B (NF-κB), adenosine monophosphate-activated protein kinase (AMPK), estrogen receptor α (ERα), CREB-binding protein, activating transcription factor 2 (ATF2), peroxisome proliferator-activated receptor (PPAR), nuclear factor (erythroid-derived 2)-like 2 (Nrf2), cyclooxygenase-2 (COX-2), and so on [59].

**Figure 1:**
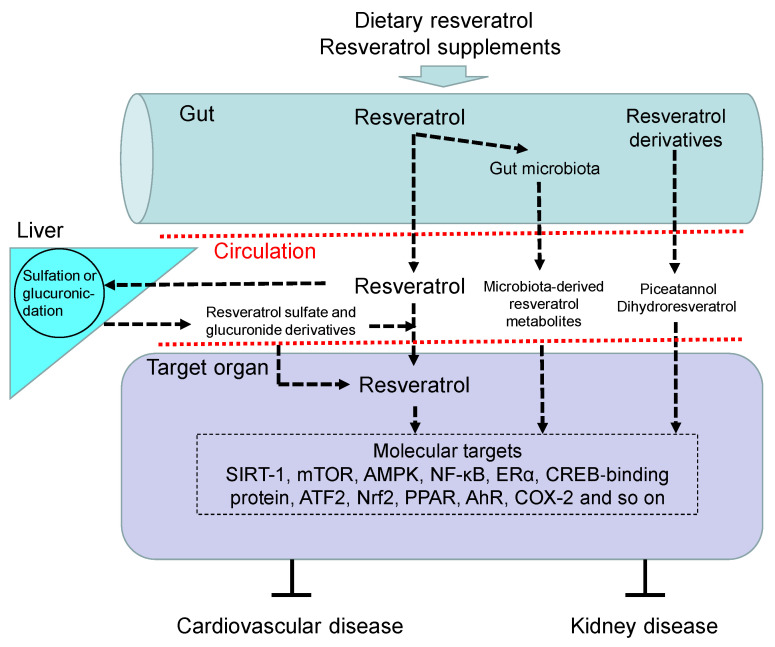
Overview of the absorption, metabolism, and targets of resveratrol. Upon oral intake, resveratrol and its precursors enter the gut and are partially metabolized by gut microbiota to produce microbiota-derived resveratrol derivatives and resveratrol. Like resveratrol, other resveratrol derivatives like piceatannol and dihydroresveratrol can also be absorbed into the blood circulation. Free resveratrol is conjugated in the liver, from where conjugated forms can return to the intestine. Resveratrol glucuronidation and sulfation in the liver to form resveratrol glucuronide and sulfate derivatives. After delivery to the target organ, resveratrol can be deconjugated to stimulate a biological response via regulation of its molecular targets, by which it benefits against cardiovascular disease and kidney disease. AhR = aryl hydrocarbon receptor. SIRT-1 = silent information regulator-1. mTOR = mammalian target of rapamycin. NF-κB = nuclear factor-kappa B. AMPK = adenosine monophosphate-activated protein kinase. ERα = estrogen receptor α. ATF2 = activating transcription factor 2. Nrf2 = nuclear factor (erythroid-derived 2)-like 2. PPAR = peroxisome proliferator-activated receptor. COX-2 = cyclooxygenase-2.

Figure 1 illustrates the absorption, metabolism, and molecular target of resveratrol, by which it benefits against CVD and kidney disease.

### 3.2. Beneficial Effects of Resveratrol in CVD

So far, only limited human trials have been conducted to examine cardiovascular benefits of resveratrol [19]. Given that that most trials evaluating combinations of resveratrol with other agents, the extent that it contributes to the combination is still ambiguous. One trial recruiting 1000 participants demonstrated that high levels of the urinary resveratrol metabolite were associated with reduced cardiovascular risk [60].

CVD is a cluster of disorders, and includes coronary heart disease, peripheral vascular disease, heart failure, congenital heart disease, cerebrovascular disease, and other conditions. In animal models, the positive effects of resveratrol have been described in a variety of CVDs, including heart failure [61], atrial fibrillation [62], myocardial ischemia-reperfusion injury [63], vascular disease [64], hypertension [65], endothelial dysfunction [66], cardiomyopathy [67], atherosclerosis [68], and stroke [69].

Several potential mechanisms mediating the protective effects of resveratrol in CVDs have been highlighted [18,21,59,64,70]. Resveratrol mainly activates SIRT-1 to augment endothelial nitric oxide synthase (eNOS) expression and improve endothelial function [63]. Additionally, resveratrol was reported to mediate Nrf2 and antioxidant response element (ARE) and exert its antioxidant function [21]. Resveratrol can also elicit anti-atherosclerotic effects, which are related to its ability to decrease the expression of adhesion molecules via inhibition of NF-κB pathway activation [71], inhibit formation of foam cells by activating Akt and forkhead box O3a (FoxO3a) pathways [72], mediate gut microbiota and its metabolites [73], inhibition of the migration and proliferation of vascular smooth muscle cells [74], and regulate lipid profile [75].

Of note is that most previous research exploring the protective actions of resveratrol on CVD has only examined the animal models of established CVD. However, whether the above-mentioned mechanisms behind beneficial actions of resveratrol are the same when applied to the models of CVD of developmental origins remains largely unclear.

### 3.3. Beneficial Effects of Resveratrol in Kidney Disease

Similar to CVD, the efficacy of resveratrol against kidney diseases is reported only in very few clinical trials [19]. The limited human studies demonstrate a protective effect of resveratrol administration on CKD patients with inconclusive results [76,77,78]. On the other hand, animal studies have shown well-proven utility of resveratrol in a number of kidney diseases, such as diabetic nephropathy [79], ischemia-reperfusion injury [80], sepsis-induced kidney injury [81], drug-induced kidney injury [82], polycystic kidney disease [83], and unilateral ureteral obstruction (UUO)-induced renal fibrosis [84].

As reviewed elsewhere [20], resveratrol can affect many signaling molecules in various kidney cells. For example, resveratrol was found to inhibit PDGF and TGF-β1 response in mesangial and epithelial cells [85,86]. Besides, several nutrient-sensing signals are molecular targets of resveratrol, such as AMPK, SIRT-1, and PGC-1α [85]. The beneficial effects of resveratrol against high glucose-induced injury on podocyte cells were associated with activation of these nutrient-sensing signals [87].

Data obtained using animal models indicate the beneficial effects of resveratrol against kidney diseases include decreasing tubulointerstitial damage and oxidative stress, reducing inflammation, increasing antioxidant activity, diminishing mesangial cell proliferation and glomeruli matrix expansion, and improving renal function [20]. As mechanisms underlying renal programming and established kidney disease are not all the same [8,11], there is an increasing need to get a better understanding of resveratrol’s reprogramming effects on the kidneys.

### 3.4. Potential Adverse Effects of Resveratrol

In humans, resveratrol shows no obvious toxicity [88,89]. Though, high dose intake of resveratrol is still related to undesired adverse events as described by some studies. One study revealed that healthy subjects were treated with multiple doses of resveratrol ranging from 25, 50, 100, to 150 mg every four hours for 48 h. Some participants developed mild adverse effects such as dizziness, headache, and epididymitis [90]. Another trial examined the safety of resveratrol at different doses (0.5, 1.0, 2.5, and 5.0 g) in healthy volunteers and found mild side effects such as diarrhea, nausea, and abdominal discomfort were noted only at the higher doses (2.5 and 5.0 g) [91]. Nevertheless, resveratrol appears to have a hormetic dose-dependent effect where resveratrol could have pro-oxidant activities at high doses, rather than antioxidant activities at low doses [92]. Hence, there is still an unmet demand to understand the optimal dosage for pregnant women to maximize the benefit of resveratrol to offspring’s cardiovascular and renal health without increasing toxicity.

## 4. Preventing CVD and Kidney Disease of Developmental Origins by Resveratrol

The World Health Organization (WHO) said global attention and urgent action are needed to prevent the increased prevalence of NCDs [93]. We, therefore, need to change strategy goal from one of risk reduction in adulthood to active intervention in early life to slow the increases in the population that develops CVD and kidney disease. Except for avoiding exposure to a suboptimal in utero conditions, another approach is to inhibit or modulate signaling pathways triggered in response to these early-life adverse experiences.

**Figure 2:**
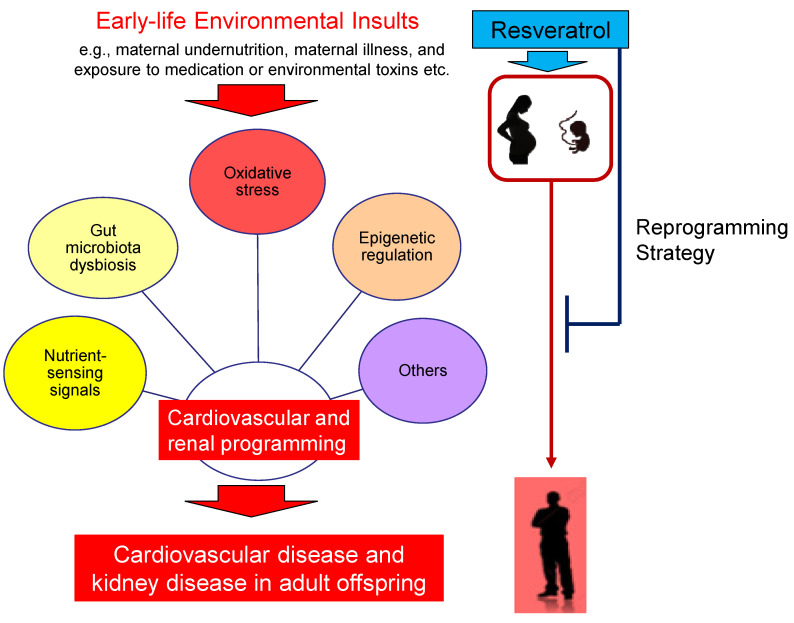
Schema outlining the cardiovascular and renal programming versus reprogramming strategy. Various early-life environmental insults can induce cardiovascular and renal programming, consequently leading to cardiovascular disease (CVD) and kidney disease in adulthood. Several common mechanisms have been proposed behind the pathogenesis of CVD and kidney disease of developmental origins, like oxidative stress, dysregulated nutrient-sensing signals, gut microbiota dysbiosis, and epigenetic regulation. Conversely, early resveratrol therapy can reverse or delay programmed processes to avoid the development of CVD and kidney disease via reprogramming.

Since resveratrol is addressed for numerous benefits to health [18,19], and it is currently used as a nutritional supplement, it is no wonder supplements of resveratrol in pregnancy have been studied to improve maternal and fetal outcome [94]. We propose a schema for summarizing the connections between early-life environmental insults, common mechanisms behind cardiovascular and renal programming, and the impact of resveratrol as a reprogramming strategy that are involved in the CVD and kidney disease of developmental origins, which is presented in Figure 2.

Currently, very limited data are available from human clinical studies regarding the effects of perinatal resveratrol supplementation on maternal influences on offspring health. There are two small studies showing that maternal resveratrol supplementation has a positive effect in pregnant women [95,96]. It is noteworthy that abnormal pancreatic development was reported in nonhuman primate offspring when exposed to resveratrol in utero [97]. However, no further study other than this group reported similar adverse outcomes.

Several species have been studied for gestational exposure of resveratrol, including rats, mice, Japanese Macaques [97], and sheep. However, only some of them reported offspring outcomes, as reviewed elsewhere [94]. Here, we summarize studies describing the reprogramming effects of resveratrol in experimental models of developmental programming, with a focus on CVD and kidney disease (Table 1) [98,99,100,101,102,103,104,105,106,107,108,109,110,111,112,113]. Resveratrol therapy will be only restricted to those starting before the onset of cardiovascular and renal phenotypes. Additionally, studies are restricted for only those offspring outcomes evaluated starting after weaning.

In the current review, limited information exists regarding the use of large animals. Table 1 shows rats are the most widely used animal models. The reprogramming effects of resveratrol supplementation have been studied in rats ranging from 3 to 20 weeks of age, which equal the developmental stages of humans, from infancy to young adulthood. Nevertheless, there is insufficient substantial data in respect to the long-term effects of resveratrol supplementation.

Various rat models of maternal insults such as a high-fat diet [98,105,106,108,111], low protein diet [99], hypoxia [100,101,102], high-fructose diet [103], adenine-induced CKD [104], N^G^-nitro-L-arginine-methyl ester (L-NAME) administration [107], combined 2,3,7,8-tetrachlorodibenzo-p-dioxin (TCDD) and dexamethasone exposure [109], or combined bisphenol A and high-fat diet [109], have been used alone or combined other postnatal insults to assess the reprogramming effects of resveratrol on the offspring’s cardiovascular and renal outcomes. Only a mouse model was reported regarding the beneficial effect of maternal resveratrol supplementation against high-fat consumption-induced obesity and hyperlipidemia [113]. Hypertension is the most common outcomes being studied [103,104,105,106,107,109,110,111,112]. Additionally, resveratrol treatment reduces several risk factors for CVD including hyperlipidemia [98,101,105,113], obesity [98,99,105,108,113], and insulin resistance [99,101].

In most studies, resveratrol was administered during pregnancy and lactation [98,99,104,105,106,107,108,109,110,112,113], a critical developmental window for organogenesis. Resveratrol was most commonly given in drinking water at the dose of 50 mg/L [98,104,105,106,107,108,109,110], followed by a chow diet supplemented with resveratrol (4 g/kg diet) [100,101,102,112]. Maternal resveratrol treatment (50 mg/L in the drinking water) attenuated hyperglycemia, obesity, and hyperlipidemia programmed by maternal high-fat intake in 3-week-old rat offspring [98]. In an adenine-induced maternal CKD rat model, resveratrol was added to drinking water during gestation and lactation and prevented male adult offspring against hypertension but had no effect on renal hypertrophy [104]. Moreover, maternal resveratrol supplementation protected adult rat offspring against hypertension induced by maternal exposure to environmental toxins, like 2,3,7,8-tetrachlorodibenzo-p-dioxin (TCDD) [109], or bisphenol A [110].

Diet supplemented with resveratrol (4 g/kg diet) improved cardiac dysfunction recovery from ischemia/reperfusion injury and attenuated insulin resistance and hyperlipidemia in rat offspring exposed to prenatal hypoxia plus postnatal high-fat diet [101,102]. In spontaneously hypertensive rats (SHR), perinatal resveratrol supplementation (4 g/kg diet) mitigated the development of hypertension in adult offspring related to enhanced NO bioavailability [112]. There exists some variability in findings that could be due to dosage, duration, and mode of administration. Hence, it suggests that the efficacy of optimal doses must be investigated and the best dosing should be determined by extensive research.

Since that various insults in gestation and lactation generate similar adverse offspring’s outcomes, and that these programming processes can be reversed or postponed by resveratrol, these observations suggest the reprogramming effects of resveratrol might mediate some common mechanisms behind the pathogenesis of CVD and kidney disease of developmental origin. Presently, cardiovascular and renal programming has been attributed to several mechanisms, including oxidative stress, dysregulated nutrient-sensing signals, aberrant renin–angiotensin-aldosterone system, gut microbiota dysbiosis, and epigenetic regulation [7,8,10,11,12,13,14,15,16,17]. Each potential mechanism linking resveratrol to cardiovascular and kidney disease of developmental origins will be discussed in detail below.

## 5. Potential Reprogramming Mechanisms of Resveratrol

### 5.1. Oxidative Stress

One of the protective mechanisms of resveratrol is its antioxidant properties [21]. The antioxidant activity of resveratrol includes reducing ROS production, inhibiting NADPH oxidase, increasing glutathione level, increasing the expression of numerous antioxidant enzymes, upregulating endothelial NOS, and increasing NO bioavailability [21].

Works that have been published in recent years support that oxidative stress is important for the developmental programming of CVD and kidney disease [7,8,10,11,12,13,14,15,16,17]. Oxidative stress is an oxidative shift characterized by an imbalance between excessive reactive oxygen species (ROS) formation and impaired antioxidant defense capacity. Although an appropriate level of ROS is essential for normal fetal development, excessively produced ROS adversely affects the developing fetus [114]. NO, a free radical and a vasodilator, also has been essential in pregnancy and fetal development. Reduced NO bioavailability as a result of inhibition by asymmetric dimethylarginine (ADMA, an NOS inhibitor) in mediating cardiovascular and renal programming has received considerable attention [12,115,116,117]. Restoration of ADMA-related ROS/NO imbalance has been considered as a reprogramming strategy to prevent developmental programming and avoid resulting CVD and kidney disease [116,117].

As illustrated in Table 1, a diversity of early-life insults has linked oxidative stress to CVD and kidney disease of developmental origins, including a high-fructose diet [103], maternal CKD [104], maternal L-NAME exposure plus postnatal high-fat diet [107], perinatal high-fat diet [108], prenatal TCDD and dexamethasone exposure [109], and maternal bisphenol A and high-fat exposure [118]. The implication of perinatal resveratrol therapy in restoration of ROS/NO balance is evidenced by the protection against hypertension in adult offspring born of dams exposed to a high-fructose diet [103], L-NAME plus postnatal high-fat diet [107], and combined BPA and high-fat exposure [110]. Moreover, resveratrol supplementation in pregnancy and lactation protects hypertension programmed by maternal CKD is associated with reduction of renal 8-hydroxy-2′-deoxyguanosine (8-OHdG, a biomarker for assessing oxidative DNA damage) expression and increases of NO bioavailability [104]. Likewise, perinatal resveratrol therapy was shown benefits against renal programming attributed to reduction of renal 8-OHdG expression, a decrease of ADMA level, and an increase of NO bioavailability in a maternal TCDD and dexamethasone exposure model [109].

### 5.2. Nutrient-Sensing Signals

Several nutrient-sensing signals belong to molecular targets of resveratrol, like AMPK, SIRT1, and PPARs [87]. Accordingly, resveratrol has been considered as a SIRT-1 or AMPK activator [18]. All aforementioned nutrient-sensing signals exist in the cardiovascular and renal systems. These signals are involved in the pathogenesis of CVD and kidney disease, which have been reviewed extensively elsewhere [118,119,120].

Nutrient-sensing signals are driven by maternal nutritional status and have a crucial role in the regulation of fetal development [121]. AMPK and SIRT-1 are able to mediate phosphorylation and deacetylation of PGC-1α, respectively [122], and consequently, to mediate the expression of PPAR target genes. PPAR target genes contributing to the pathogenesis of renal programming and programmed hypertension include *Nos2*, *Nos3*, *Sod2*, *Nrf2*, *Sirt7*, *Ren*, and *Sgk1* [123]. As early-life nutritional insults can dysregulate nutrient sensing signals to mediate PPARs target genes [124], nutrient-sensing signaling might therefore be a common mechanism behind CVD and kidney disease of developmental origins [125,126]. Conversely, early-life interventions targeting AMPK signaling has been deemed as a reprogramming strategy to prevent developmental origins of hypertension [127].

In a maternal L-NAME plus high-fat diet model [107], resveratrol therapy protected adult offspring against hypertension and coincided with activation of AMPK/SIRT1/PGC-1α pathway. Additionally, our prior research revealed that AMPK activation prevents the elevation of offspring’s BP via regulation of nutrient-sensing signals in models of developmental hypertension programmed by a high-fat diet [111] and a high-fructose diet [103]. These observations support the notion that the interplay between resveratrol and nutrient-sensing signals are implicated in CVD and kidney disease of developmental origins.

### 5.3. Gut Microbiota Dysbiosis

With a prebiotic effect for gut microbes, the beneficial effects of resveratrol are also related to its ability to alter gut microbiota [128,129]. Gut microbiota derived metabolites can affect the function of various target organs through circulation, including the cardiovascular and renal systems [130,131,132]. Several mechanisms have been proposed behind CVD and kidney disease attributed to gut microbiota dysbiosis, including alterations of short-chain fatty acids (SCFA) and tryptophan-derived metabolites, increases of trimethylamine-N-oxide (TMAO), increased sympathetic activity, inhibition of NO, and aberrant activation of the RAS [130,131,132].

Maternal insults can alter the offspring’s gut microbial composition, leading to consequent adverse offspring outcomes [133]. On the other hand, maternal microbiota-targeted interventions have shown benefits against cardiovascular and renal programming [134,135,136]. Our prior research demonstrated that supplementation with prebiotic inulin, probiotics *Lactobacillus casei*, or postbiotics acetate during gestation and lactation can protect adult offspring against hypertension programmed by various early-life insults [134,135,136].

Using a high-fructose model [103], we previously found that resveratrol therapy protected adult offspring against programmed hypertension related to alterations of gut microbiota, particularly increased the proportions of *Lactobacillus* and *Bifidobacterium*, two well-known probiotic strains. Likewise, resveratrol therapy in gestation and lactation protected adult offspring against hypertension programmed by maternal CKD, which was associated with increased abundance of *Lactobacillus* and *Bifidobacterium* as well as increased microbial richness and diversity [104]. Moreover, the protective effects of resveratrol against hypertension of developmental origins may also relate to its ability to reduce the *Firmicutes* to *Bacteroidetes* ratio, a microbial marker for hypertension [130,131], in a maternal L-NAME plus high-fat diet [107]. Thus, it is speculated that resveratrol may act as a prebiotic by reshaping the gut microbiome and promoting the growth of beneficial microbes to reprogram CVD and kidney disease of developmental origins.

### 5.4. Epigenetic Regulation

As we mentioned earlier, resveratrol can activate SIRT-1, as SIRT-1 is an NAD^+^-dependent deacetylase, making resveratrol one of the earliest nutraceuticals with associated epigenetic activity [137]. In addition to deacetylation, resveratrol can modulate epigenetic patterns by directing the enzymes that catalyze histone modifications and DNA methylation or altering levels of S-adenosylmethionine, the key donor in methylation reactions [138].

Epigenetic regulation is another important mechanism behind developmental programming [139]. Maternal exposure to lipopolysaccharide (LPS) results in hypertension in adult rat offspring and is associated with increases in global DNA methylation level in offspring kidneys [140].

Aberrant DNA methylation in several genes belonging to the renin-angiotensin system (RAS), a well-known pathway involved in BP control, has been linked to hypertension of developmental origins [141]. Another epigenetic mechanism is post-translational modifications to histone proteins. Histone post-translational modification, mediated by histone acetyltransferases (HATs) and deacetylases histone acetylation (HDACs), is one of the most studied factors affecting gene expression. HDACs were found to regulate expression of several genes in the RAS, including angiotensinogen, renin, angiotensin-converting enzyme (ACE), and angiotensin type 1 receptor (AT1R) [142]. Moreover, microRNAs (miRNAs), the most commonly studied small non-coding RNAs (ncRNA) [143], are also implicated in epigenetic processes of the RAS elements in renal programming and programmed hypertension [144]. A recent study reported that grape juice promotes specific miRNA-mediated cardioprotection against myocardial infarction in a mouse model [145]. These data suggest that resveratrol-mediated miRNAs may be a potential reprogramming strategy for CVD of developmental origins and future studies are warranted. Although resveratrol constitutes functional foods and cardioprotective role of epigenetic compounds delivered by functional foods has been proposed [146], little reliable information currently exists with regard to epigenetic effects detected in clinical cardiovascular trials of functional foods with a focus on resveratrol.

HDAC and DNA methyltransferase (DNMT) inhibitors have shown benefits against hypertension programmed by prenatal dexamethasone exposure [147,148]. It is noteworthy that resveratrol has been considered as an HDAC as well as a DNMT inhibitor [149]. However, little information currently exists regarding the reprogramming effects of resveratrol related to its epigenetic activity. Only one report revealed that resveratrol therapy prevented adult offspring against obesity and is associated with epigenetic regulation of leptin and its receptor through DNA methylation [108]. There will be a growing demand to better understand the epigenetic mechanisms of resveratrol on CVD and kidney disease of developmental origins, and to be able to develop ideal reprogramming interventions.

### 5.5. Others

As far as the multifaceted actions of resveratrol, there are other potential mechanisms by which resveratrol might motivate: (1) by activating Nrf2, and (2) by antagonizing AhR. Although Nrf2 activation has been shown to be of benefit in other models of developmental programming [150,151], there are not enough data to conclude what the reprogramming effects of resveratrol with programmed CVD and kidney disease are directly through regulation of Nrf2. Additionally, resveratrol has been reported as an AhR antagonist [152]. During development, exposures to environmental chemicals can increase the risk of CVD in adulthood [153]. These chemicals, like TCDD and BPA, are known as ligands for AhR [154]. Of note is that resveratrol therapy prevented TCDD- or BPA-induced hypertension of developmental origins was linked to mediation of AhR signaling pathway [109,110]. These findings suggest there might be an interplay between resveratrol and AhR behind the pathogenesis of hypertension of developmental origins, although this remains speculative.

Resveratrol acting like a mind-boggling cornucopia benefits CVD and kidney disease of developmental origins. Although several mechanisms are outlined above, attention will need to be paid to explore other potential mechanisms. Better understanding interaction between resveratrol and protective mechanisms are key toward developing ideal reprogramming interventions for further clinical translation.

## 6. Conclusions and Perspectives

Current evidence in support of the protective role of resveratrol therapy in CVD and kidney disease of developmental origins is strong but incomplete. One major unsolved problem limiting therapeutic applications of resveratrol is due its low bioavailability in vivo [53,57]. Presently, various methods have been established to enhance its bioavailability [155,156]. Nevertheless, these resveratrol-related compounds have not been examined in vivo yet, especially in CVD and kidney disease of developmental origins.

Another important aspect is that significant progress has been made in elucidating beneficial effects of resveratrol in established CVD and kidney disease, while less attention has been paid to its reprogramming effects in developmental programming. Due to its diverse biological activities, the reprogramming mechanisms of maternal resveratrol therapy are difficult to predict. Additional attention will need to be paid to get a full-scope view of various reprogramming mechanisms and test its dose-dependency in a diversity of developmental programming models to maximize the benefit without increasing toxicity. Even though growing evidence from animal studies supporting resveratrol therapy as a reprogramming strategy to protect against CVD and kidney disease of developmental origins, these results are still awaiting clinical translation into human subjects.

## Figures and Tables

**Table 1:** Eligible animal studies reporting offspring outcomes related to CVD and kidney disease after resveratrol supplementation.

Species/Gender	Animal Models	Dose and Duration	Age at Evaluation	Offspring Outcomes	Ref.
Wistar ras/M & F	Maternal high-fat diet	Resveratrol (50 mg/L) in drinking water during gestation and lactation	3 weeks	Attenuated hyperglycemia, obesity and hyperlipidemia	[98]
Wistar rat/M & F	Maternal low protein diet	Resveratrol (20 mg/kg/day) via oral gavage during gestation	16 weeks	Attenuated obesity and insulin resistance	[99]
SD rat/M	Prenatal hypoxia and postnatal high-fat diet	Resveratrol (4 g/kg of diet) between 3 to 12 weeks of age	12 weeks	Improved cardiac tolerance to ischemia	[100]
SD rat/M	Prenatal hypoxia and postnatal high-fat diet	Resveratrol (4 g/kg of diet) between 3 to 12 weeks of age	12 weeks	Attenuated insulin resistance and hyperlipidemia	[101]
SD rat/M & F	Prenatal hypoxia and postnatal high-fat diet	Resveratrol (4 g/kg of diet) between 3 to 12 weeks of age	21 weeks	Improved cardiac dysfunction recovery after ischemia/reperfusion (I/R) injury	[102]
SD rat/M	Maternal plus post-weaning high-fructose diet	Resveratrol (50 mg/L) in drinking water from weaning to three months of age	12 weeks	Prevented hypertension	[103]
SD rat/M	Maternal chronic kidney disease	Resveratrol (50 mg/L) in drinking water during gestation and lactation	12 weeks	Prevented hypertension No effect on renal hypertrophy	[104]
SD rat/M	Maternal plus post-weaning high-fat diet	Resveratrol (50 mg/L) in drinking water during gestation and lactation	16 weeks	Prevented obesity, hypertension, and hyperlipidemia	[105]
SD rat/M	Maternal plus post-weaning high-fat diet	Resveratrol (50 mg/L) in drinking water during gestation and lactation	16 weeks	Attneuated hypertension	[106]
SD rat/M	Maternal L-NAME administration plus post-weaning high-fat diet	Resveratrol (50 mg/L) in drinking water during gestation and lactation	16 weeks	Prevented hypertension	[107]
SD rat/M	Maternal plus post-weaning high-fat diet	Resveratrol (50 mg/L) in drinking water during gestation and lactation	16 weeks	Prevented obesity	[108]
SD rat/M	Maternal TCDD and dexamethasone exposure	Resveratrol (0.05%) in drinking water during gestation and lactation	16 weeks	Prevented hypertension	[109]
SD rat/M	Maternal exposure to Bisphenol A and high-fat diet	Resveratrol (50 mg/L) in drinking water during gestation and lactation	16 weeks	Prevented hypertension	[110]
SD rat/M	Maternal plus post-weaning high-fat diet	0.5% resveratrol in drinking water between 2 to 4 months of age	16 weeks	Prevented hypertension	[111]
SHR/M & F	Genetic hypertension	Resveratrol (4 g/kg of diet) during gestation and lactation	20 weeks	Mitigated hypertension	[112]
C57BL/6 J mouse/M	Maternal plus post-weaning high-fat diet	0.2% *w*/*w* resveratrol in diet during gestation and lactation	14 weeks	Prevented obesity and hyperlipidemia	[113]

Studies tabulated according to species, animal models, and age at evaluation; SD rats = Sprague-Dawley rats; M = male; F = female; TCDD = 2,3,7,8-tetrachlorodibenzo-p-dioxin; L-NAME = N^G^-nitro-L-arginine-methyl ester.
